# Intraoperative and postoperative management in endoscopic ear surgery: Perspectives from a worldwide survey

**DOI:** 10.1007/s00405-026-10485-5

**Published:** 2026-08-03

**Authors:** Cecilia Lotto, Jessica Bertolasi, Alice Bernardocchi, Ignacio Javier Fernandez, Lukas Anschuetz, Giulia Molinari

**Affiliations:** 1 Department of Otorhinolaryngology, Head and Neck Surgery, CHUV, University of Lausanne, Lausanne, Switzerland; 2 The Sense Innovation and Research Center, Lausanne and Sion, Lausanne, Switzerland; 3 U-VIP Unit for Visually Impaired People, Fondazione Istituto Italiano di Tecnologia, Genoa, 16163 Italy; 4 Department of Informatics, Bioengineering, Robotics and Systems Engineering (DIBRIS), Genoa, 16145 Italy; 5 Alma Mater Studiorum - University of Bologna, Bologna, Italy; 6 Department of Otorhinolaryngology, Ospedale Santa Maria delle Croci, Ravenna, 48121 Italy; 7 Department of Otolaryngology-Head and Neck Surgery, IRCCS Azienda Ospedaliero-Universitaria of Bologna, Bologna, Italy

**Keywords:** Endoscopic ear surgery, Antibiotic prophylaxis, Dry ear, Suppurative ear, Ear packing, Questionnaire

## Abstract

**Purpose:**

To examine intraoperative and postoperative practices (antibiotic and antithrombotic prophylaxis, ear packing, behavioural recommendations, follow-up strategies) in exclusive endoscopic ear surgery management, based on a global survey of experienced surgeons.

**Methods:**

The setting is an international survey distributed via personal mailing lists and the International Working Group on endoscopic ear surgery. Anonymous responses were collected through Google Forms and analyzed using descriptive statistics, logistic regression models, and Kruskal-Wallis tests with post-hoc analyses to explore associations between geographic origin, surgical practices, and postoperative protocols. The result is a cross-sectional descriptive study.

**Results:**

We collected responses from 44 surgeons across 21 different countries. Significant variability in surgical management practices was observed, often diverging from the current literature. Geographic origin and surgeon experience showed a limited association with specific practices. Notably, North American surgeons reported longer follow-up durations and more consistent use of cefazolin, whereas European surgeons demonstrated greater variability in antibiotic regimens.

**Conclusion:**

The survey reveals wide variability in the intraoperative and postoperative management of exclusive endoscopic ear surgery, driven more by individual preferences than by scientific evidence. The absence of consistent patterns based on geography or expertise highlights the lack of standardized, evidence-based protocols.

**Supplementary Information:**

The online version contains supplementary material available at https://doi.org/10.1007/s00405-026-10485-5.

## Introduction

Endoscopic ear surgery (EES) has seen a significant rise in popularity over the past decade, demonstrating its value as a standalone technique or as an adjunct to conventional microscopic surgery. Enhanced visualization, reduced invasiveness, and improved cosmetic outcomes have contributed to its success [1–3]. A growing body of robust evidence on surgical and functional outcomes - often in comparison to the microscopic technique - has revealed the safety and efficacy of EES [4–7]. Despite its increasing use, critical aspects of perioperative management have not yet been the focus of rigorous research or scientific debate [8–11].

Antibiotic prophylaxis is considered essential to prevent surgical site infections, a concern heightened by the potential for contamination and the possible negative impact on post-operative healing. However, unnecessary use contributes to resistance, and studies since 1980 suggest that prophylaxis is unnecessary in clean ear surgery [12–14]. Yet, no consensus exists on the best regimens for contaminated surgeries, as evidenced by significant worldwide variability [10, 15]. Similarly, antithrombotic prophylaxis is crucial for preventing deep vein thrombosis (DVT) and other thromboembolic events, but guidelines specific to this minimally-invasive technique, which generally involves short operative times, are lacking [10]. Moreover, despite being widely practiced, ear packing techniques vary significantly across centers, and the ideal material has yet to be identified. Finally, also postoperative care, including at-home recommendations, wound care, and follow-up schedules, varies widely among practitioners [16].

To explore approaches to perioperative and postoperative EES management, we created and distributed a comprehensive survey to endoscopic ear surgeons worldwide. By collecting detailed data on intra- and postoperative practices, this study aims to provide a global snapshot of EES management strategies. These insights may foster a collaborative move toward more standardized, evidence-based practice in otology.

## Materials and methods

In May 2023, we developed an English questionnaire targeting otologic surgeons worldwide, focusing on intra- and postoperative management in exclusive EES (defined as procedures performed entirely with an endoscopic technique, without the use of a microscope at any stage). The survey included eight sections:

1. “Your Experience”: country of practice and number of EES surgeries performed annually.
2. “Intraoperative Prophylaxis”: use, route, specific drug(s) and dosage of intraoperative antibiotic prophylaxis, and whether it varied by surgery type.
3. “Ear Packing”: materials used to pack the EAC, and whether they were soaked with drugs.
4. “DVT Prophylaxis”: whether postoperative prophylaxis is performed, the criteria for its indication, and the dosage administered.
5. “Postoperative Antibiotic Therapy in a Dry Ear”: topical, systemic, or combined antibiotic therapy in cases of non-infected ears, defined as ears without active discharge and previously treated with appropriate medical therapy prior to surgery.
6. “Postoperative Antibiotic Therapy in a Suppurative Ear”: antibiotic treatment practices in cases with intraoperative evidence of infection, defined as the presence of active otorrhea and inflamed middle-ear mucosa, indicating a wet ear with exudate, regardless of the presence of cholesteatoma.
7. “Postoperative Care”: recommendations given to patients after surgery (e.g., water protection, avoiding air travel or heavy lifting) and timing of ear packing removal.
8. “Follow-Up”: timing of postoperative follow-ups, dressing removal, and audiological assessments, including bone conduction testing before discharge.

The questionnaire was formatted using Google Forms and distributed via a unique link embedded in an email describing the study (Supplement 1). Participants were contacted via email through personal mailing lists and the International Working Group on Endoscopic Ear Surgery (IWGEES), including all members (*n* = 193). A reminder email was sent in September to increase participation. A total of 44 responses were received (response rate: 22.8%), and all submitted questionnaires were complete. All participants consented to participate to the survey and this study was conducted in accordance with the Declaration of Helsinki (2013). Data were collected anonymously and recorded solely for the purposes of this research. The processing of data complied with applicable legislation governing the use of such data for scientific research purposes.

Data were collected from November 2023 to July 2024 and analyzed using Microsoft Excel and MATLAB R2024 [17]. Descriptive statistics summarized the survey results. Binary and multinomial logistic regression models explored associations between factors such as continent of origin or EES volume and specific clinical practices. To investigate differences in postoperative recommendations across continents, we performed a Kruskal-Wallis test, followed by post-hoc comparisons to identify which groups significantly differed.

## Results

Data were collected from 44 surgeons across 21 different countries. The majority were from Europe (23/44,52%), followed by Asia(12/44,27%), North America(6/44,14%), Oceania(2/44,5%) and Africa(1/44,2%). 8 surgeons(18%) performed less than 40 EES procedures per year, 16 (36%) performed 40–70 surgeries, 9(20%) between 70 and 99 and 11(24%) more than 100 interventions annually.

### Intra-operative practice

**Fig. 1:**
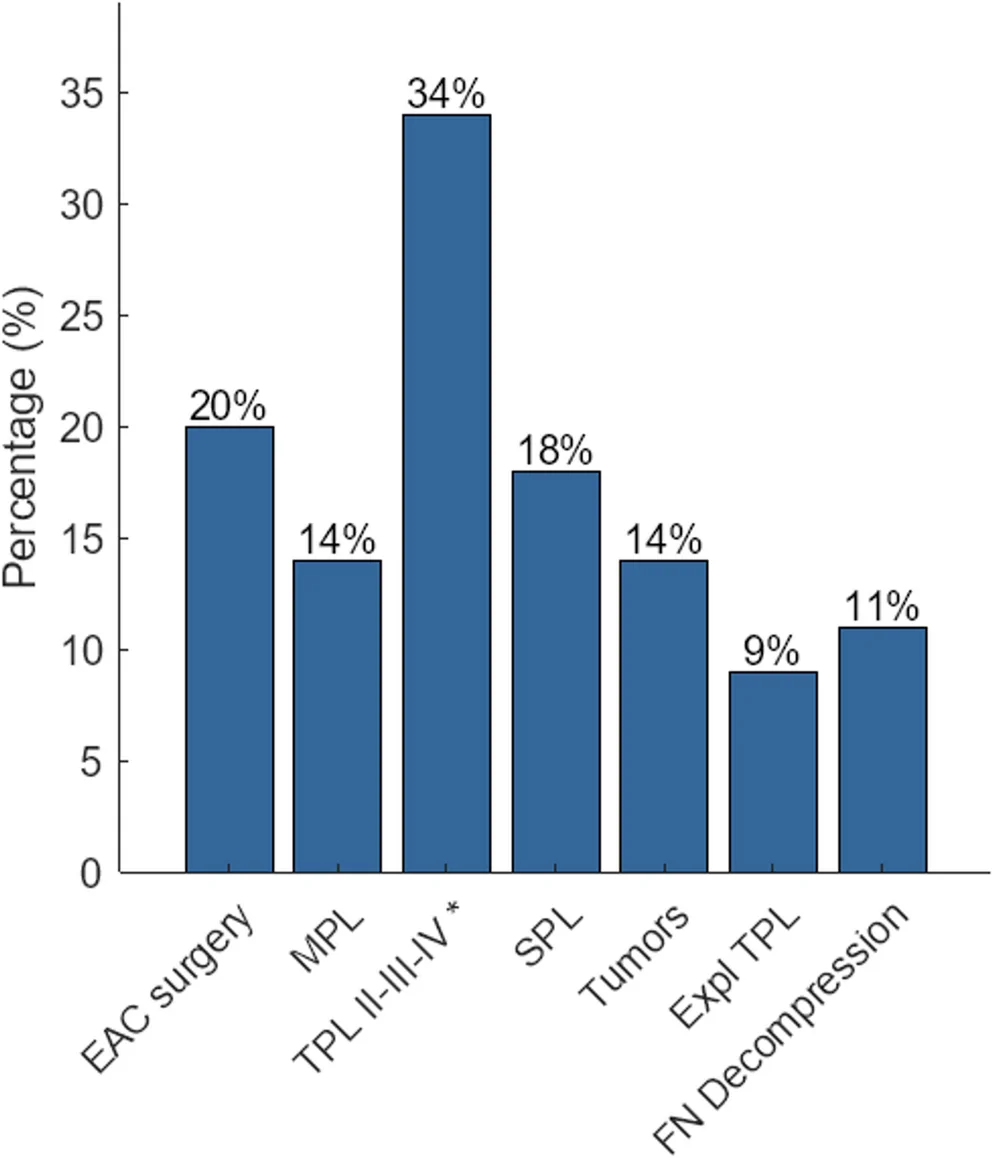
Distribution of intraoperative antibiotic prophylaxis by surgery type, based on responses from surgeons who selectively prescribe antibiotics. EAC: external auditory canal; MPL: myringoplasty; TPL: tympanoplasty, SPL: stapedoplasty; Expl: exploratory

Among the surveyed surgeons, only 25 (57%) reported using intraoperative antibiotic prophylaxis. Notably, none of the surgeons from Oceania reported administering intraoperative prophylaxis. All surgeons administering intraoperative prophylaxis preferred the intravenous route. However, only 8 surgeons (18%) applied antibiotic prophylaxis in all EES cases, while the remaining 17 (39%) reserved its use for specific ear surgeries. (Fig. 1).

A binary logistic regression was performed to assess the association between continent of origin and the administration of intraoperative prophylaxis. Results are presented in Table 1. as odds ratios (OR) with 95% confidence intervals (CI). The model, which estimates the probability of not administering prophylaxis, showed no statistically significant effect for any of the continents compared to the reference group (Europe). The intercept was not significant (β = −0.44, SE = 0.43, *p* = 0.30), and none of the variables indicating the different continents showed a significant association (*p* > 0.63 for all comparisons, except for Africa and Oceania, for which the estimate is unreliable due to numerical problems in convergence).

**Table 1:** Estimates for each continent presented as odds ratios (OR) with 95% confidence intervals (CI)

Continent	OR, 95% CI	*p*-value
Europe	0.64, [0.28, 1.49]	0.30
Africa	NE	1.00
North America	1.56, [0.26, 9.47]	0.63
Asia	1.11, [0.27, 4.60]	0.88
Oceania	NE	1.00

No statistically significant associations were observed for any continent compared to Europe. For North America and Asia, the estimated ORs indicate no clear evidence of an association and substantial uncertainty due to wide confidence intervals. Estimates for Africa and Oceania were not reliable due to quasi-complete separation, resulting in non-estimable OR and infinite CI. *NE* = not estimable due to quasi-complete separation and model instability

A binary logistic regression was conducted to evaluate the association between the number of surgeries performed and the administration of intraoperative prophylaxis. No statistically significant associations between the number of EES and the analyzed outcome were found, with estimated odds ratios close to 1 and accompanied by wide 95% confidence intervals, suggesting limited precision and no clear evidence of an effect (see Table 2).

**Table 2:** Estimates for each number of surgeries performed presented as odds ratios (OR) with 95% confidence intervals (CI)

Number of EES	OR, 95% CI	*p*-value
< 40	0.60, [0.14, 2.51]	0.48
40–70	2.14, [0.38, 12.20]	0.39
71–99	1.33, [0.19, 9.31]	0.77
> 99	0.63, [0.09, 4.40]	0.64

No statistically significant associations were observed. All confidence intervals were wide and included the null value (OR = 1), indicating substantial uncertainty and no clear evidence of an association

**Fig. 2:**
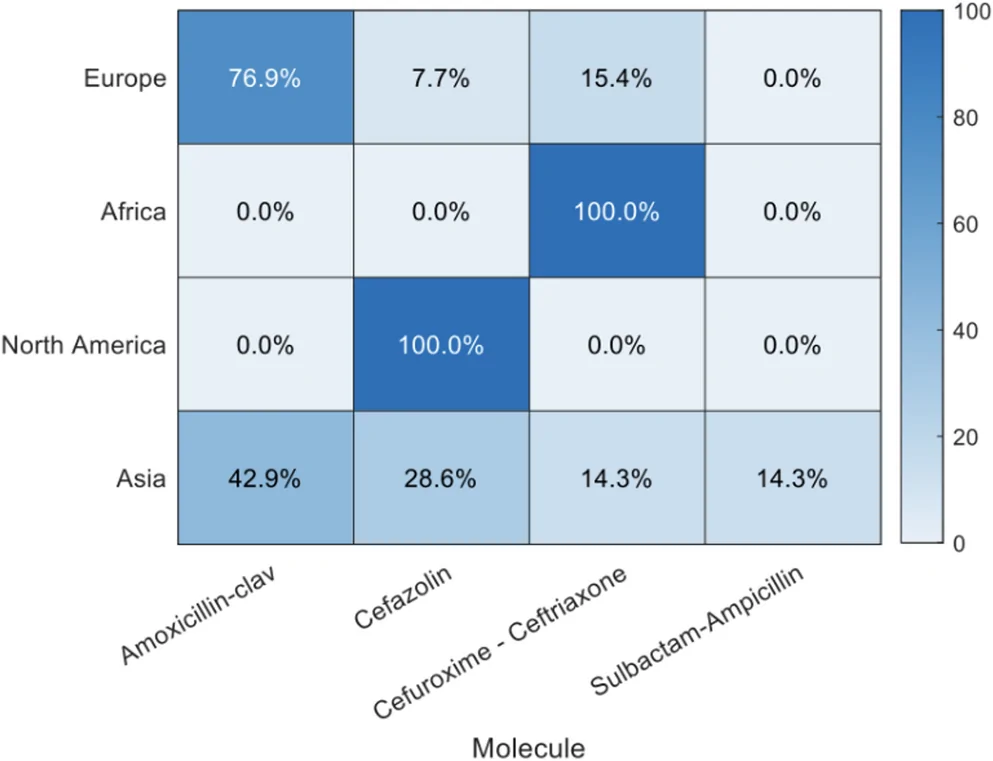
Percentage distribution of antibiotic molecule usage across different continents. Rows represent continents, and columns represent distinct molecules. The color intensity corresponds to the proportion of molecules used within each continent (darker shades indicating higher percentages)

All surgeons who reported using intraoperative prophylaxis administered the same antibiotic across different EES procedures. A heatmap (Fig. 2) shows the distribution of preferences for antibiotic molecules.

### Ear packing after surgery

All surgeons involved in this study performed EAC packing after surgery. The data revealed a preference for absorbable sponge alone(25, 57%) as the primary packing material, as illustrated in Table 3. Nevertheless, according to a multinomial logistic regression, there is no evidence of a statistically significant relationship between continents and the type of packing used.

**Table 3:** Preferences of different packing materials among the respondents

Type of packing material	*N*° of surgeons using the specific packing material
Absorbable sponge only	25 (57%)
Silk and absorbable sponge	6 (14%)
Silastic and absorbable sponge	5 (11%)
BIPP ± absorbable sponge	5 (12%)
Merocel^®^ ± absorbable sponge	3 (6%)

*BIPP* Bismuth Iodoform Paraffin Paste

**Fig. 3:**
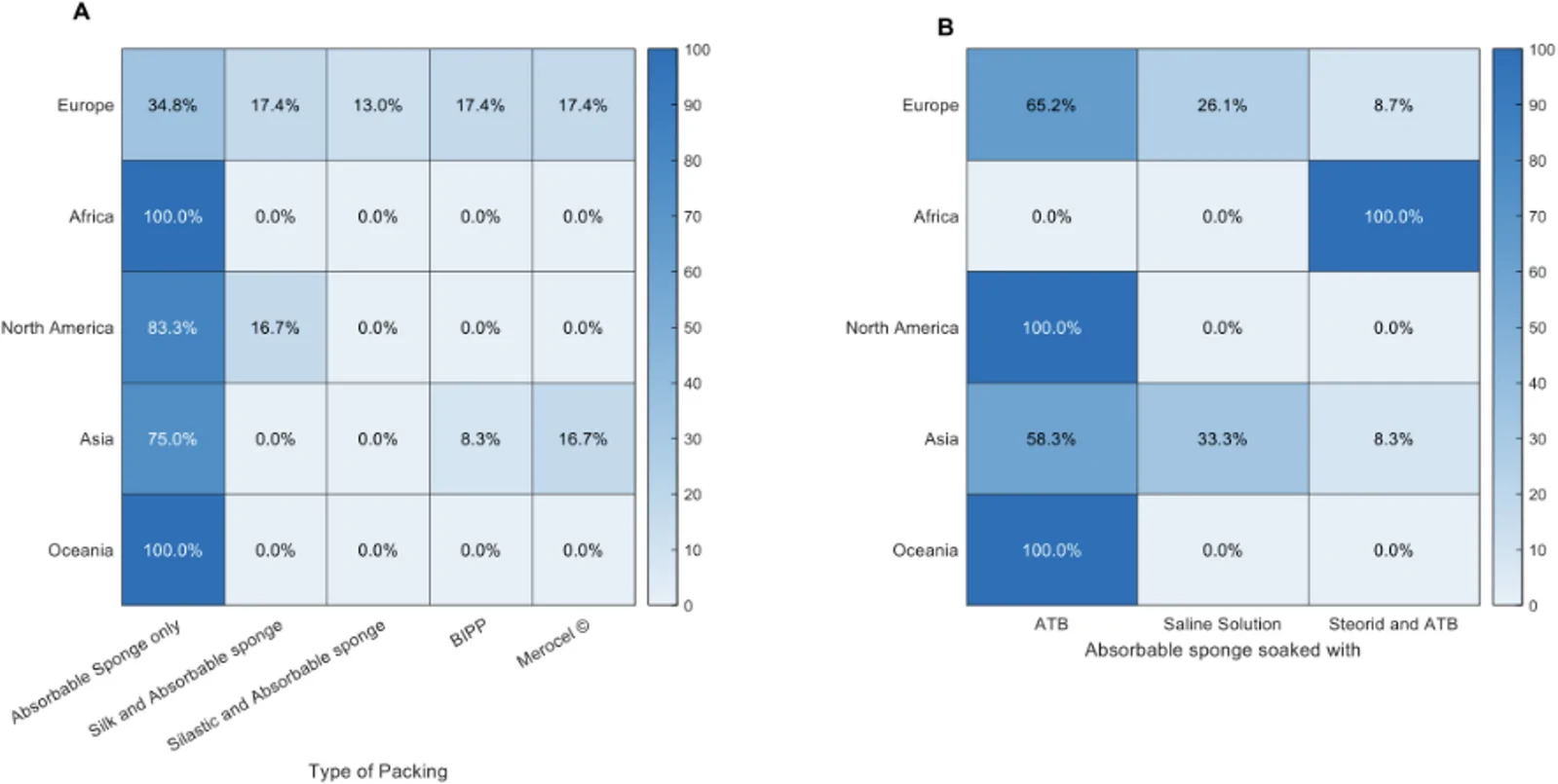
Percentage distribution of post-surgery packing materials used (**A**) and specific molecule used to soak the Absorbable Sponge (**B**) across continents. Each row corresponds to a continent and each column to a packing type for absorbable sponge

On additional treatments applied to the sponges, the findings revealed a clear preference (30/44, 68%) for antibiotics. Table 4 and Fig. 3 detail the antibiotic preferences.

**Table 4:** Number of surgeons using a specific antibiotic molecule in the packing material

Type of antibiotic used in packing material	*N*° of surgeons using the antibiotic
Ciprofloxacin	11 (25%)
Ofloxacin	7 (16%)
Doxycycline	3 (7%)
Polymyxin B	3 (7%)
Fucidin (Fusidic Acid)	2 (4%)
Ofloxacin + Bactroban (mupirocin)	1 (2%)
Vibramicine	1 (2%)
Not specified	6 (14%)

Ten surgeons(23%) opted to soak the absorbable sponge with saline solution only, while a smaller number(4/44,9%) combined antibiotics with steroids (50% use of dexamethasone, 25% Hydrocortisone and 25% variable molecules).

### Short-term post-operative management

#### Deep vein thrombosis prophylaxis

Only 2 surgeons(4.5%) always used DVT prophylaxis, while 9 (20.5%) never used it.

**Fig. 4:**
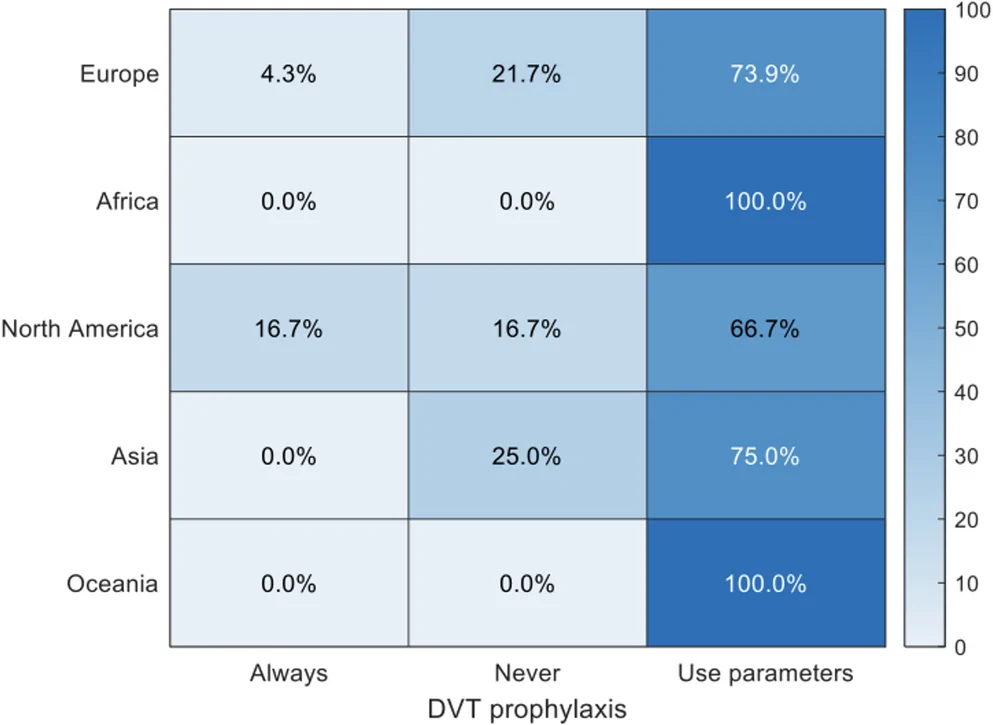
Heatmap illustrating the choices reported by surgeons on DVT prophylaxis with respect to continents of origin

The heatmap in Fig. 4 indicates that all continents demonstrate a distinct preference regarding the selection of parameters.

The majority (33/44 surgeons,75%) based their decisions on patient-specific parameters. The duration of prophylaxis ranged from 1 to 7 days.

Age was considered a parameter for DVT prophylaxis by 9 surgeons. Common cut-offs were 65 years (2/44 surgeons) and 60 years (3/44). BMI was a decision-making factor for DVT prophylaxis according to 13 surgeons, with common cut-offs varying between 30 (4/44), 35 (2/44), 25 (1/44), 23 (1/44). The most used parameter (21/44, %) was duration of surgery. Common cut-offs were > 1 h (4/44 surgeons), 45 min (4/44), 3 h (2/44), 4 h (1/44). Caprini Score was only used by 3 surgeons. Decision-making in cases of thrombophilia was referred to hematologic consultation by 2 surgeons. Mechanical compression as a prophylactic measure was mentioned twice.

#### Post-operative antibiotic treatment

Indications for post-operative antibiotic use were evaluated based on the infectious status observed during surgery (“dry ear” vs. “suppurative ear”). For dry ear cases, 55% of surgeons (24/44) did not prescribe postoperative antibiotics, while 45% (20/44) did. In suppurative ear cases, most surgeons (37/44,84%) used postoperative antibiotics as shown in Table 5. The most commonly chosen systemic drug was Amoxicillin-clavulanate, followed by cephalosporins and fluoroquinolones. Only one respondent reported prescribing antibiotics based on antibiogram results. Conversely, the choice of local therapy is less varied, with fluoroquinolones being the preferred option.

**Table 5:** Specific antibiotic molecules used as systemic and local therapy in dry and suppurative ears, respectively

Dry ear	Number of surgeons	Type of drugs (number of surgeons)	Duration of therapy (mean, days ± standard deviation)
Local	10	- Ciprofloxacin (3) - Ofloxacin (4) - Ciprofloxacin + Dexamethasone (3)	11 ± 5.5
Systemic	7	- Amoxicillin-clavulanate (4) - Ceftriaxone (1) - Amoxicillin-clavulanate + Ceftriaxone (1) - Cefuroxime (1)	5.8 ± 2.5
Local + Systemic	3	**Local**: Ciprofloxacin (3) **Systemic**: - Ciprofloxacin (2) -Amoxicillin-clavulanate (1)	Local: 11.7 ± 8.1 Systemic: 11.7 ± 8.1
Suppurative ear	**Number of surgeons**	**Type of drugs (number of surgeons)**	**Duration of therapy (mean**,** days ± standard deviation)**
Local	11	- Ofloxacin (3) - Ciprofloxacin (4) - Ciprofloxacine + Dexamethasone (2) - Oxitetracicline + Hydrocortisone + Polimixine B (1) - Ciprofloxacine + Hydrocortisone (in MPL/TPL) - Tobramicine + Dexamethasone (in others surgeries) (1)	8.6 ± 4.3
Systemic	15	- Amoxicillin-clavulanate (10) - Ciprofloxacine (1) - Ceftriaxone (1) - Amoxicillin-clavulanate + Ceftriaxone (1) - Ceftriaxone in SPL - Sultamicilline in MPL/TPL (1) - According to antibiogram (1)	6.5 ± 2.3
Local + Systemic	11	**Local**: - Ciprofloxacine (7) - Ciprofloxacine + Dexamethasone (1) - Ofloxacine (1) - Tobramicine + Dexamethasone (1) - No answer (1) **Systemic**: - Amoxicillin-clavulanate (5) - Ciprofloxacine (4) - Cefuroxime (1) - Cefuroxime + Ceroxime (1)	Local: 12.2 ± 5.7 Systemic: 8.3 ± 4.8

*Atb* antibiotic

**Fig. 5:**
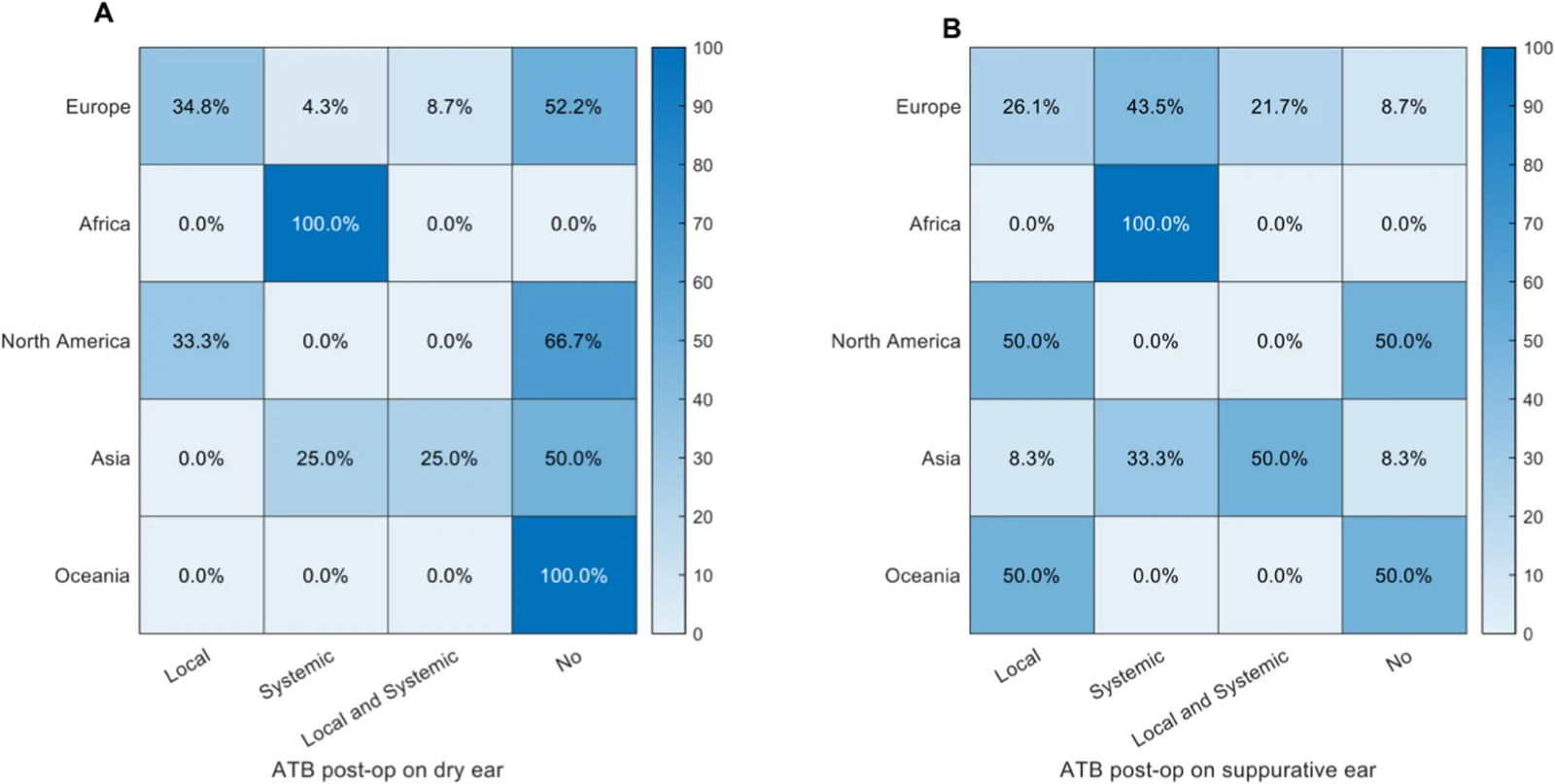
Heatmaps illustrating the distribution of responses regarding the use of postoperative antibiotic therapy in dry (**A**) or suppurative (**B**) ears, categorized by continent

For both dry and suppurative ears, no significant association was found between the use of postoperative antibiotics and continent of origin or the number of EES performed annually in multinomial logistic regression analyses. Figure 5 presents a distribution of the responses provided by participants regarding the treatment for dry or suppurative ear, categorized by continent.

### Long-term post-operative management

#### At-home recommendations

Surgeons provided various postoperative recommendations following exclusive EES, with durations specified for both stapes and other procedures. To assess whether the duration of post-operative recommendations differed between continents, the Kruskal-Wallis test was used for each of the recommendations. None of the analyses revealed statistically significant differences between the geographical groups (*p* > 0.05) for either stapedotomy or other interventions.

**Fig. 6:**
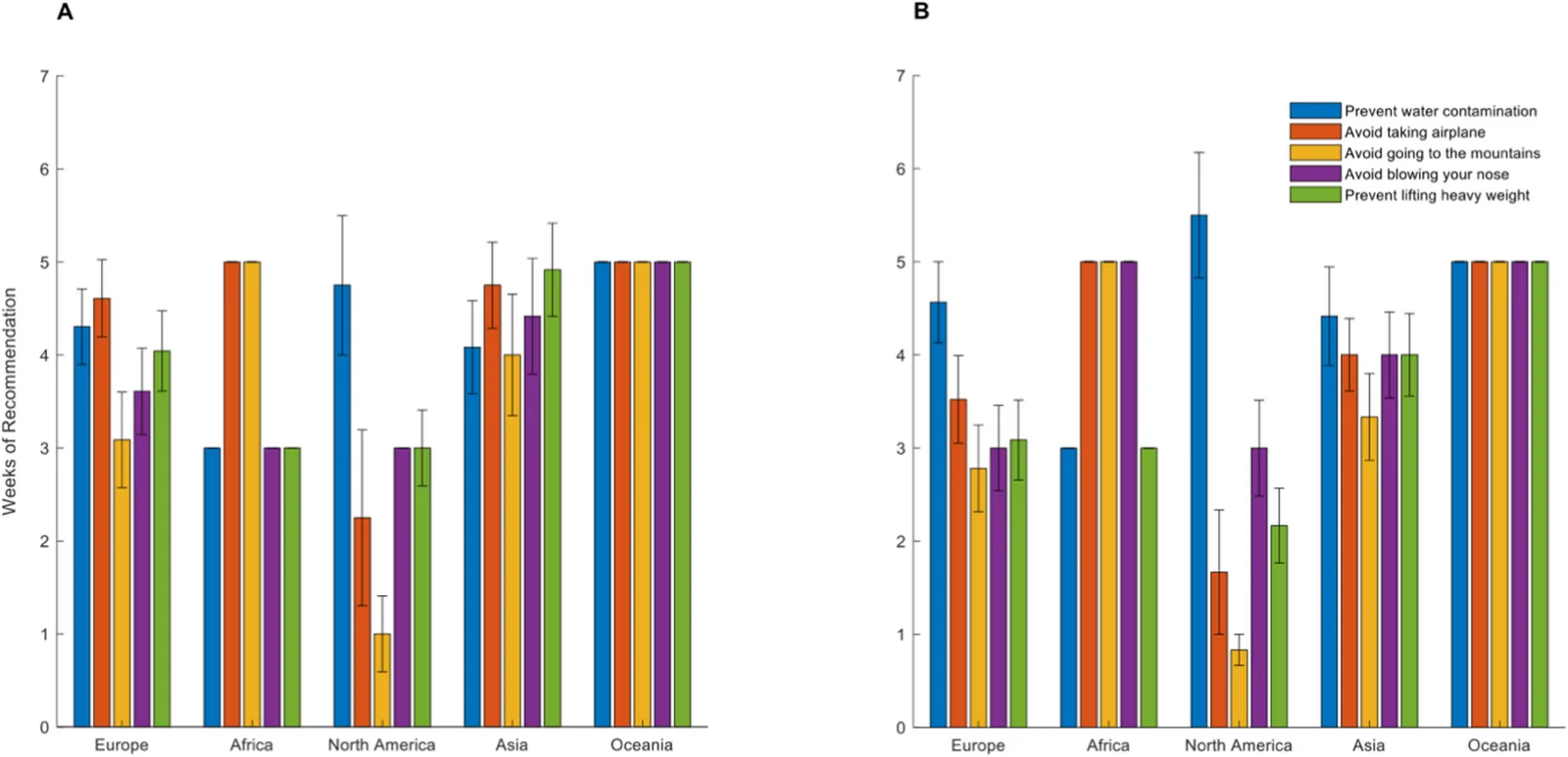
Recommended average duration (weeks) to abstain from specific activities in the post-operative period according to the continent the participant belongs to, specifically in panel A, post-stapedotomy surgery, and in panel B, for all other surgeries

As summarized in Fig. 6 for stapes procedures, patients were generally advised to avoid water contamination for an average of 4.26 weeks. Additionally, they were recommended to refrain from going to the mountains for an average of 3.26 weeks and avoid air travel for an average of 4.45 weeks. Only one surgeon did not suggest avoiding air travel at all, while 8 recommended 8 weeks of suspension. To minimize pressure changes, patients should avoid blowing their nose for approximately 3.81 weeks and refrain from weightlifting for an average of 4.21 weeks.

Patients undergoing other than stapes surgery were advised to prevent water contamination for an average of 4.61 weeks, avoid air travel for approximately 3.48 weeks, and refrain from going to the mountains for 2.80 weeks. They should avoid blowing their nose for about 3.39 weeks and avoid weightlifting for an average of 3.27 weeks.

#### Follow-up

Thirty-two surgeons(73%) removed ear packing after an average of 14.6 days (range 5–37), while 12(27%) waited for spontaneous resorption. The binary logistic regression model assessing the relationship between continent of origin and packing removal did not show any significant association. The first follow-up is performed within the first 10 days by 20 surgeons(45.5%), between the 10th and 14th day by 9(20.5%), and after 14 days by the remaining 15(34%). The average follow-up is performed 12 days after surgery. The second follow-up is carried out within the first month by 20 surgeons(45%), within 3 months by 18 surgeons (41%), after the third month by 6 surgeons (14%), with 2 surgeons conducting the second follow-up at 1 year.

Kruskal-Wallis test, associating the continent of origin and the number of days to the first follow-up, showed a statistically significant difference between the groups(H(4) = 16.82, *p* = 0.0021), indicating that time to first visit varies by continent. Post-hoc analysis with non-parametric multiple comparisons revealed that participants from North America had significantly longer times than those from Asia (*p* = 0.002), as well as the European surgeon(*p* = 0.04). The same analysis was performed concerning the days to the second post-operative assessment, with the test showing statistically significant differences between the groups (H(4) = 14.66, *p* = 0.0055), and in particular, the post-hoc analysis showed a significant difference between North American and Asian participants (*p* = 0.006), with North American participants receiving their first assessment significantly later. Other comparisons between groups did not reach statistical significance. It is important to notice that given the small sample size and uneven geographic representation of the partecipants, these intercontinental comparisons should be considered exploratory.

The Kruskal–Wallis test revealed no statistically significant differences in the timing of the follow-ups defined by the number of operations performed by surgeons, indicating that high-volume surgeons do not tend to delay follow-up compared to lower-volume surgeons.

Regarding the audiological follow-up, a postoperative bone conduction test before discharge was performed by 15 doctors, with no significant difference between continents (*p* > 0.05).

A complete audiological exam was performed by nine surgeons within 30 days, by 29 within 3 months, and by three surgeons after 3 months. Three surgeons (7%) do not perform a complete audiological exam during follow-up, but only the Weber test.

## Discussion

This study reports the current intraoperative and postoperative practices of otologic surgeons worldwide when performing exclusive EES. The answers from the 44 surgeon, along with the statistical analysis, suggest a widely variable approach to the management of perioperative antibiotic and antithrombotic prophylaxis, as well as ear packing and postoperative follow-up strategies.

### Antibiotic prophylaxis

Fear of middle ear infections drives antibiotic use both intra- and postoperatively. Studies evaluating the role of antibiotic prophylaxis in ear surgery date back to the 1980 s and have consistently suggested that it provides no significant benefit in tympanoplasty and myringoplasty since then. Several studies, including those involving large patient cohorts and trials on chronic otitis media, found no reduction in infection or graft failure rates, nor any improvement in surgical success or audiometric outcome [12–14]. Similar results were confirmed by a Cochrane review, updated in 2009, including 11 randomized controlled trials [8].

To address bias from pooling clean and contaminated cases, Govaerts et al. conducted a randomized controlled trial distinguishing between “clean” and “dirty” ears, finding higher infection risk in cases with cholesteatoma and draining perforations. They reported that using intraoperative cefuroxime in this group led to a threefold reduction in infection [18]. A 2012 review of five prospective, double-blind, randomized studies similarly showed lower infection rates with targeted intraoperative antibiotics for draining cholesteatoma [19]. Despite this, the contamination classification of the surgical site remains complex. A Cochrane review categorized common surgeries (tympanostomy tube placement, tympanoplasty, and mastoidectomy) as clean procedures [8]. However, procedures involving cholesteatoma or infected ears are inconsistently labeled as clean-contaminated or contaminated, complicating standardization. This inconsistency remains a major obstacle in establishing standardized antimicrobial guidelines, as the baseline infectious risk associated with different surgical types may influence postoperative outcomes. The lack of well-controlled studies on the effectiveness of local and systemic antibiotics in preventing surgical infections facilitates variability in surgical practices, as highlighted by our survey.

According to our results, most surgeons (25/44,57%) used intravenous prophylaxis, mainly Amoxicillin-clavulanate, with 17 of them (68%) limiting it to specific surgeries (mostly tympanoplasty, including cholesteatoma surgery). This practice contrasts with literature and raises concerns about unnecessary antibiotic exposure, costs, and resistance, one of the most pressing global health challenges of the 21 st century [15]. Postoperatively, 45% of surgeons prescribed antibiotics for dry ears, indicating overuse. Conversely, 84% treated suppurative ears using systemic, local, or combined regimens. Statistical analyses confirmed a lack of consensus, attributed to the absence of strong evidence-based guidelines.

A 2023 survey comparing Austrian and Canadian surgeons revealed no consensus on antibiotic use for endoscopic stapes and tympanoplasty. Austrian surgeons, like those in the present study, showed wide variability and mostly prescribed Amoxicillin-clavulanate, while Canadian surgeons preferred Cefazolin [15]. This difference reflects broader guidelines, which recommend Cefazolin for procedures not involving the mucosa of the aerodigestive tract and Amoxicillin-clavulanate when the mucosa is breached, relevant when *Staphylococcus aureus* is a concern. The ear represents a unique anatomical region, as the external ear is not part of the aerodigestive tract, while the middle ear is lined with respiratory epithelium, making standardization more complex. Moreover, the mean duration of postoperative antibiotics in our survey was 5,8 days, exceeding the generally recommended 24–48-hour window [20].

An American survey also noted that many surgeons prescribe antibiotics based on habit rather than evidence [21], highlighting the persistent gap between literature and real-world practice.

### Ear packing

In our survey, all participating surgeons perform ear packing routinely, with more than half using exclusively absorbable materials, reflecting a strong preference across all continents. Despite its widespread use, no consensus exists on optimal material or technique. Ear packing materials can cause complications such as retraction, granuloma, or transient hearing loss. Ideal packing would balance support, absorption, cost, and biocompatibility, yet no universally accepted material fulfills all these criteria. Absorbable options like Gelfoam may increase infection risk, while other options (e.g., fibrin glue) are promising but not standardized [22, 23].

A randomized trial by Sahoo et al. found comparable outcomes between absorbable and non-absorbable materials, with only transient hearing differences. Similarly, there is no consensus on whether packing should be soaked in antibiotics or when it should be removed [24]. A 2020 study comparing removal at 2 vs. 4 weeks suggested delayed removal may support healing [25], although most surgeons in this survey remove packing around day 15. An emerging question in the literature is whether ear packing is necessary at all. Early studies suggest omitting packing does not negatively impact surgical outcomes and may reduce patient discomfort and follow-up needs, though more robust evidence is required [11, 26, 27].

### Deep vein thrombosis prophylaxis

EES procedures typically involve young, healthy patients and relatively short operative times, making DVT uncommon. Despite this, many surgeons still use heparin prophylaxis, basing their decisions primarily on a history of thrombosis (26 respondents) and the duration of surgery (21 respondents). However, the threshold for defining “long surgery” varied from 45 min to 4 h. There is minimal recent literature supporting antithrombotic use in otologic surgery. A 1976 study found no cases of DVT in ear surgery patients. Given the absence of robust data, no consensus guidelines exist for DVT prevention in EES [10].

### At-home recommendations

Postoperative behavior guidance also varied widely. Stapes surgery is particularly sensitive to pressure changes, prompting varying recommendations regarding air travel and diving. Surveys from 1996 to 2016 showed no agreement, with advice ranging from no restriction to six-month delays [28, 29].

A 2023 analysis by Akhal and Bassim explained pressure dynamics during flights and suggested that safe Eustachian tube opening during descent mitigates risks [30]. Hüttenbrink’s cadaveric study demonstrated that even large pressure changes cause only minor prosthesis displacement, further suggesting that flying post-stapedotomy is likely safe [16]. Recommendations to avoid altitude or water exposure after ear surgery are also inconsistent. A 2007 survey of 195 ENT consultants found no agreement on water precautions, and existing data remain inconclusive [31]. This underscores the need for clear, standardized postoperative guidance based on clinical evidence.

### Follow-up

**Fig. 7:**
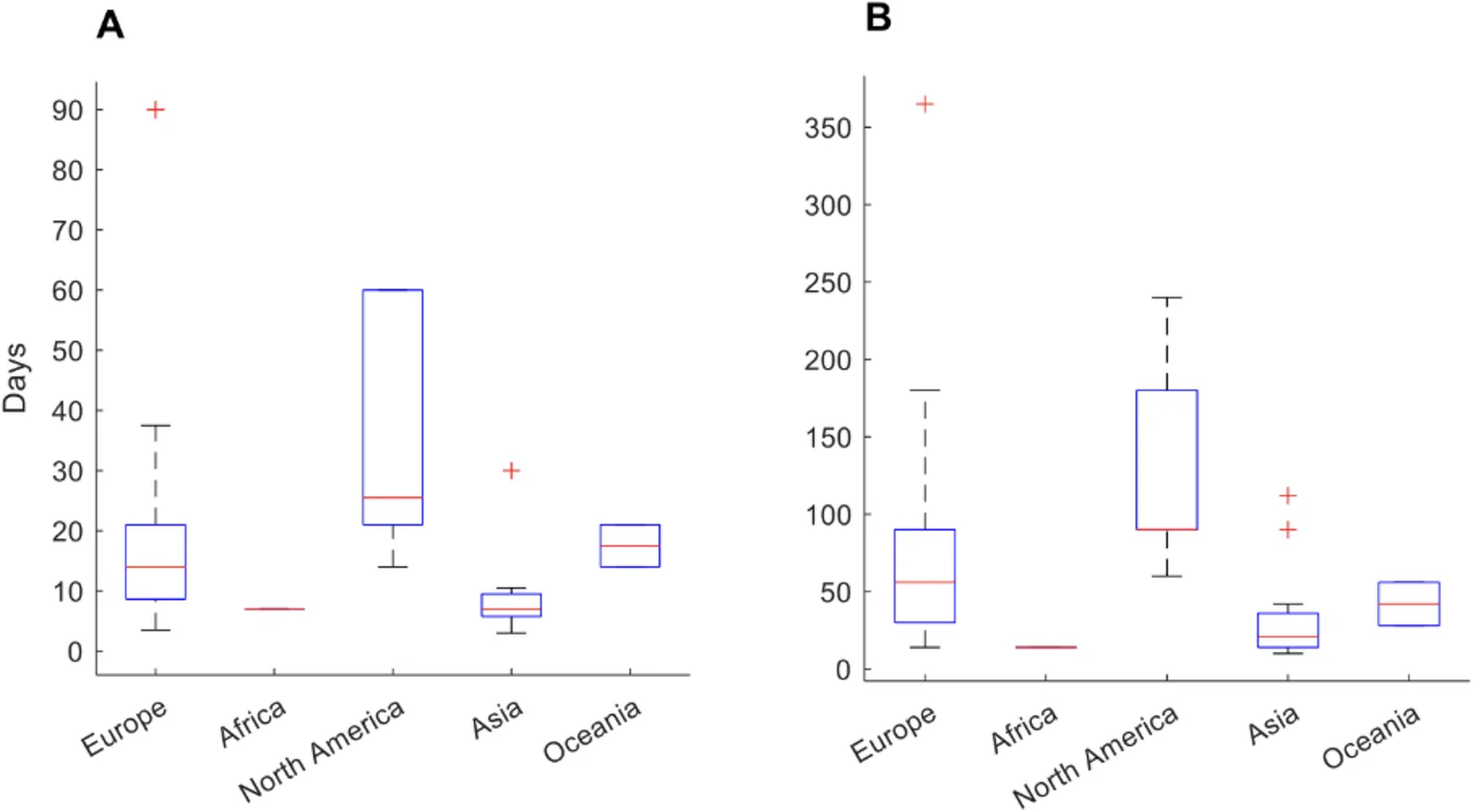
Distribution of days to first (**A**) and second (**B**) post-operative assessment for each continent to which participants belong. The skewness of distributions and the presence of outliers support the use of non-parametric tests for statistical analysis

In our study, 65% of respondents do not recommend a bone conduction test prior to discharge. We found no evidence in the literature supporting the necessity of this test to assess inner ear integrity postoperatively. While the Rinne and Weber tests have established sensitivity and specificity, their reliability diminishes in cases of bilateral hearing loss [32, 33]. Most surgeons (66%) conduct full audiometric testing 2–3 months postoperatively, likely waiting for packing to resorb and reduce test interference. A minority (*n* = 9) conducts audiometry at one month, typically after earlier packing removal. We observed regional differences in postoperative management. European surgeons tended to remove packing earlier than others (*p* = 0.002), while North American surgeons scheduled both first and second follow-ups significantly later than their Asian counterparts Fig. 7.

## Limitations of the study

This study has several limitations. Although surgeons from multiple continents were included, the number of respondents was relatively small and the distribution of responders’ origin was uneven, with a predominance of European participants, considering the possibility of selection bias resulting from recruitment through personal networks. This may have reduced the sensitivity of intercontinental comparisons.

As a survey-based study, the results rely on self-reported data and are therefore subject to reporting bias. Reported practices may reflect habitual or intended behavior rather than actual clinical management in all cases. In addition, responses could not be verified against clinical records. Furthermore, the absence of formal pilot testing and external validation represents a methodological limitation of the study. In the context of an international survey encompassing participants from diverse training backgrounds and healthcare systems, this may have introduced variability in question interpretation and could potentially affect the internal consistency and reproducibility of the responses.

Differences in national healthcare systems, institutional policies, and medico-legal frameworks likely influenced perioperative management decisions, including antibiotic use, DVT prophylaxis, and follow-up timing. Variations in pathogen prevalence or resistance profiles between regions may reasonably lead surgeons to favor broader-spectrum antibiotics or to extend the duration of treatment. These systemic factors were not controlled for and may account for part of the observed variability.

Finally, the study was descriptive and did not assess clinical outcomes or stratify responses by disease severity or patient comorbidities. Consequently, the findings should be interpreted as a snapshot of current practice rather than evidence supporting specific management strategies.

## Conclusion

This survey explores multiple perioperative aspects of exclusive EES and reveals a pronounced degree of heterogeneity in clinical practice. The wide variation in surgeons’ approaches appears to stem largely from the absence of consistent, evidence-based. Indeed, no meaningful associations were found between surgical practices and either geographic location or procedural volume.

This study reports only practice patterns and cannot establish the superiority of any approach.

Observed differences, such as longer postoperative follow-up in North America and variation in antibiotic strategies, including more consistent cefazolin use compared with Europe, should be interpreted cautiously given the small sample size and uneven geographic distribution, particularly the limited representation from some regions. Nonetheless, they provide important insights into current global practices in EES.

Collectively, these findings highlight the urgent need for robust, outcome-based comparative studies to determine which perioperative strategies are most effective, safe, and resource-efficient, and to support the development of standardized, evidence-based guidelines for this increasingly utilized technique.

## Supplementary Information

Below is the link to the electronic supplementary material.

Supplementary Material 1 (DOCX 18.8 KB)

## Data Availability

The datasets generated and/or analyzed during the current study are available from the corresponding author on reasonable request.
