# Supplementary material for: Intraoperative and postoperative management in endoscopic ear surgery: Perspectives from a worldwide survey
Source: Eur Arch Otorhinolaryngol. 2026 Aug 3;283(11):7065–75. doi: 10.1007/s00405-026-10485-5 (PMC13748167; doi:10.1007/s00405-026-10485-5)
Supplement: Supplementary file 1 [file 405_2026_10485_MOESM1_ESM.docx]

**Intraoperative and postoperative management in endoscopic ear surgery: perspectives from a worldwide survey**

Cecilia Lotto^1,2^, Jessica Bertolasi^3,4^, Alice Bernardocchi^5^, Ignacio Javier Fernandez^5,6^, Lukas Anschuetz^1,2^, Giulia Molinari^5,7^

^1^ Department of Otorhinolaryngology, Head and Neck Surgery, CHUV, University of Lausanne, Lausanne, Switzerland.

^2^ The Sense Innovation and Research Center, Lausanne and Sion, Lausanne, Switzerland.

^3^ U-VIP Unit for Visually Impaired People, Fondazione Istituto Italiano di Tecnologia, Genoa, 16163, Italy

^4^ Department of Informatics, Bioengineering, Robotics and Systems Engineering (DIBRIS), Genoa, 16145, Italy

^5^ Alma Mater Studiorum - University of Bologna, Bologna, Italy.

^6^ Department of Otorhinolaryngology, Ospedale Santa Maria delle Croci, 48121 Ravenna, Italy.

^7^ Department of Otolaryngology-Head and Neck Surgery, IRCCS Azienda Ospedaliero-Universitaria of Bologna, Bologna, Italy.

**Corresponding Author**

Alice Bernardocchi, MD

Department of Otolaryngology-Head and Neck Surgery, IRCCS Azienda Ospedaliero-Universitaria of Bologna, Bologna, Italy.

Email: alice.bernardocchi@studio.unibo.it

**SUPPLEMENT 1.**

**YOUR EXPERIENCE**

1. In which country do you work as an ear surgeon?
2. How many totally endoscopic ear surgery procedures do you perform per year (considering the last five years)?
   - I do not perform totally endoscopic ear surgery at all
   - Less than 40
   - 40-70
   - 71-99
   - More than 100

**INTRAOPERATIVE PROPHYLAXIS**

3. Do you use intraoperative prophylaxis in any of your EES?
   - Yes
   - No

**EAR PACKING**

4. What type of packing do you apply to the external auditory canal at the end of a totally EES procedure?
   - Absorbable sponge only
   - Silk or (artificial silk) and absorbable sponge
   - Sylastic and absorbable sponge
   - Other
5. The absorbable sponge you use is:
   - Soaked in saline solution
   - Filled with steroid
   - Filled with antibiotic
   - Filled with steroid AND antibiotic
6. If you use steroid and/or antibiotic, please specify the molecule and dose
7. Is there any specific EES procedure at the end of which you use a different ear packing? If yes, please specify

**DEEP VEIN THROMBOSIS PROPHYLAXIS**

8. What are the criteria to indicate deep vein thrombosis prophylaxis in EES cases at your institution? Please specify the cut off for each criteria.
   - Age
   - BMI
   - Duration of surgery
   - Previous thrombosis
   - Other
9. For how many days after surgery d you prescribe deep vein thrmbosis prophylaxis?

**POST-OPERATIVE ANTIBIOTIC THERAPY IN DRY EAR**

10. Do you use any antibiotic therapy in the post-operative period after totally EES on a DRY EAR?
    - No
    - Yes, local antibiotic therapy
    - Yes, systemic antibiotic therapy
    - Both local and systemic antibiotic therapy
11. Do you use the same molecule and dose of local antibiotic for every procedure in a DRY ear?
    - Yes
    - No
12. Please specify which molecule and dose of local antibiotic you prescribe after each surgical procedure in a DRY ear
13. Please specify how long is the local antibiotic therapy after any EES procedure (number of days) in a DRY ear
14. Do you use the same molecule and dose of systemic antibiotic for every procedure in a DRY ear?
    - Yes
    - No
15. Please specify which molecule and dose of systemic antibiotic you prescribe after each surgical procedure in a DRY ear
16. Please specify how long is the systemic antibiotic therapy after any EES procedure (number of days) in a DRY ear

**POST-OPERATIVE CARE**

17. Which recommendations do you give to your patients after totally EES STAPES SURGERY and for how long?
    - Prevent water contamination
    - Avoid taking the airplane
    - Avoid going to the mountains
    - Avoid blowing your nose
    - Prevent lifting heavy weights
18. Which recommendations do you give to your patients after totally EES (OTHER THAN STAPES SURGERY) and for how long?
    - Prevent water contamination
    - Avoid taking the airplane
    - Avoid going to the mountains
    - Avoid blowing your nose
    - Prevent lifting heavy weights
19. Do you remove the dressing of the external auditory canal?
    - Yes
    - No

**FOLLOW-UP**

20. When do you plan the first post-operative follow up (days after surgery)?
21. When do you plan the second post-operative follow up (days after surgery), if the first was regular?
22. Do you perform a formal post-operative bone conduction hearing test before discharging the patient?
    - Yes
    - No
23. When do you perform a complete post-operative pure tone audiometry to assess postopoerative hearing function? (days after surgery)
